# Supplementary material for: A scoping review protocol to map the evidence on interventions to prevent overweight and obesity in children
Source: BMJ Open. 2018 Feb 14;8(2):e019311. doi: 10.1136/bmjopen-2017-019311 (PMC5829943; doi:10.1136/bmjopen-2017-019311)
Supplement: Supplementary file 1 [file bmjopen-2017-019311supp001.pdf]

Supplementary material 1

Search strategy

CINAHL Plus via EBSCOhost

**Date of search**	18/08/2017
**Search string**	(TI (obesity OR overweight OR weight) OR MH (obesity OR pediatric obesity)) AND (TI (scoping OR systematic OR meta OR review) OR MH (review OR meta-analysis)) AND (AB (child* OR adolescen* OR you*) OR MH (child OR adolescent)) AND AB (prevention OR intervention)
**Number of results**	178 for AB; 185 for SU
**Number of results**	363

Cochrane Library of Systematic Reviews

**Date of search**	18/08/2017
**Search string**	MeSH descriptor: [Pediatric Obesity] explode all trees
**Number of results**	12

ERIC

**Date of search**	26/07/2017
**Search string**	(title:(obesity OR overweight OR weight) AND (title:(scoping OR systematic OR meta OR review)) AND (abstract:(child* OR adolescen* OR you*))
**Number of results**	454

ERIC via EBSCOhost

**Date of search**	26/07/2017
**Search string**	TI (obesity OR overweight OR weight) AND TI (scoping OR systematic OR meta OR review) AND AB (child* OR adolescen* OR you*) AND AB (prevention OR intervention)
**Number of results**	35

Google Scholar

**Date of search**	26/07/2017
**Search string**	(obesity OR overweight OR weight) (scoping OR systematic OR meta OR review) (child OR adolescen OR you) (prevention OR intervention)<br>allintitle: (obesity OR overweight OR weight) (scoping OR systematic OR meta OR review) (child OR adolescen OR you)
**Number of results**	104

Joanna Briggs Institute (JBI) Database of Systematic Reviews and Implementation Reports via OVID

**Date of search**	28/07/2017
**Search string**	(Ti: ((obesity OR overweight OR weight) AND (scoping OR systematic OR meta OR review))) AND (ab:((child* OR adolescen* OR you*) AND (prevention OR intervention))
**Number of results**	31

MEDLine/PubdMed MeSH

**Date of search**	18/08/2017
**Search string**	((((obesity[Title] OR overweight[Title] OR weight[Title] OR obesity[MeSH Terms] OR pediatric obesity[MeSH Terms])) AND (scoping[Title] OR systematic[Title] OR meta[Title] OR review[Title] OR Review[MeSH Terms] OR Meta-analysis[MeSH-Terms])) AND (child*[Title/Abstract] OR adolescen*[Title/Abstract] OR you*[Title/Abstract] OR Child[MeSH Terms] OR Adolescent[MeSH Terms])) AND (prevention[Title/Abstract] OR intervention[Title/Abstract])
**Number of results**	792

NHS EED

**Date of search**	28/07/2017
**Search string**	((obesity OR overweight OR weight)):TI OR ((scoping OR systematic OR meta OR review)):TI OR ((child* OR adolescen* OR you*)):TI AND ((prevention OR intervention)):TI
**Number of results**	99

PsycINFO via EBSCOhost

**Date of search**	28/07/2017
**Search string**	TI (obesity OR overweight OR weight) AND TI (scoping OR systematic OR meta OR review) AND AB (child* OR adolescen* OR you*) AND AB (prevention OR intervention)
**Number of results**	275

Scopus

**Date of search**	28/07/2017
**Search string**	TITLE(obesity OR overweight OR weight) AND TITLE(scoping OR systematic OR meta OR review) AND ABS(child* OR adolescen* OR you*) AND ABS(prevention OR intervention)

**Number of results**	501

SocINDEX via EBSCOhost

**Date of search**	28/07/2017
**Search string**	TI (obesity OR overweight OR weight) AND TI (scoping OR systematic OR meta OR review) AND AB (child* OR adolescen* OR you*) AND AB (prevention OR intervention)
**Number of results**	20

Web of Science / Web of Knowledge

**Date of search**	18/07/2017
**Search string**	TI=((obesity OR overweight OR weight) AND (scoping OR systematic OR meta OR review)) AND TS=((child* OR adolescen* OR you*) AND (prevention OR intervention))
**Number of results**	567
