# Supplementary figures and images for: A scoping review protocol to map the evidence on interventions to prevent overweight and obesity in children
Source: BMJ Open. 2018 Feb 14;8(2):e019311. doi: 10.1136/bmjopen-2017-019311 (PMC5829943; doi:10.1136/bmjopen-2017-019311)

## Supplementary material 2

### PRISMA flowchart

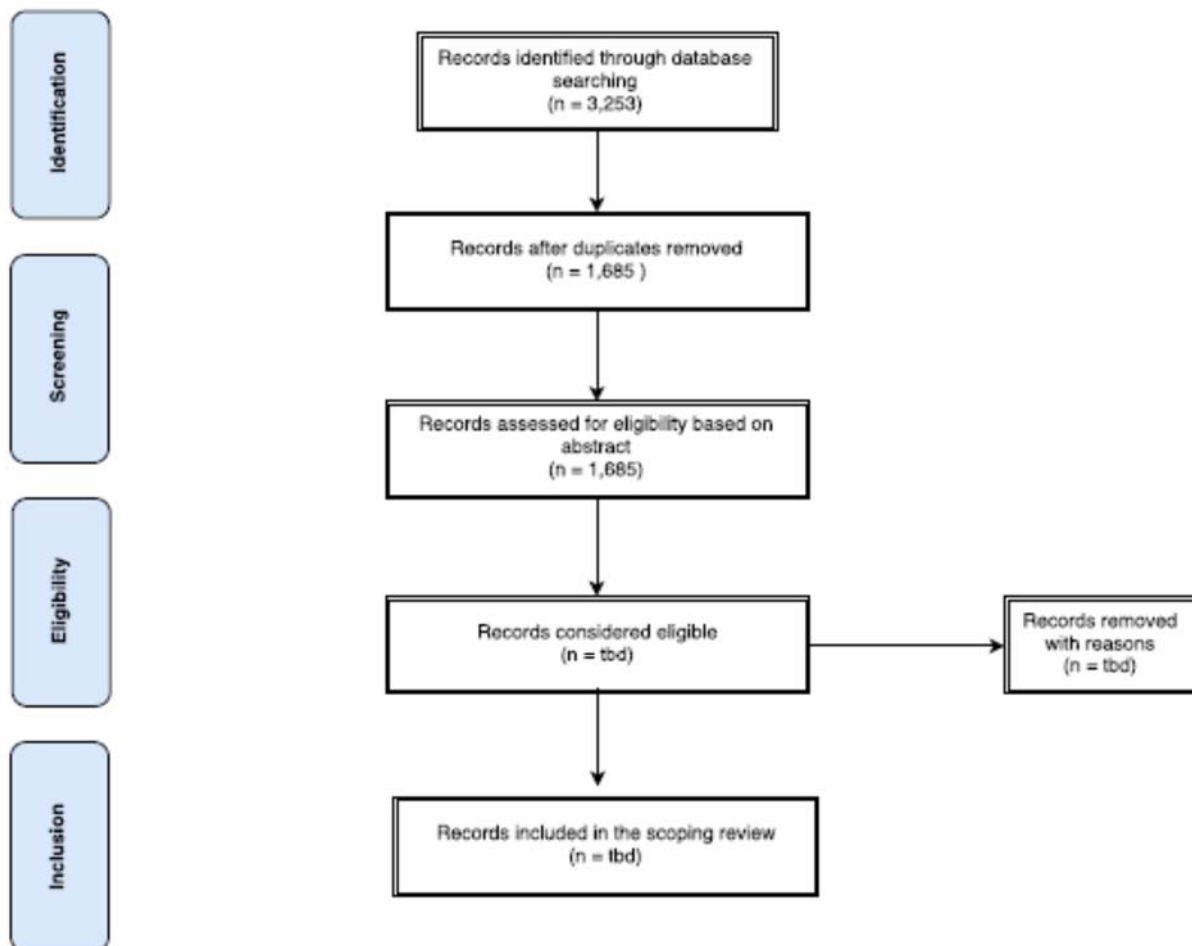

Supplement: Supplementary file 2 [file bmjopen-2017-019311supp002.pdf]
