# Supplementary material for: A scoping review protocol to map the evidence on interventions to prevent overweight and obesity in children
Source: BMJ Open. 2018 Feb 14;8(2):e019311. doi: 10.1136/bmjopen-2017-019311 (PMC5829943; doi:10.1136/bmjopen-2017-019311)

BMJ Open is committed to open peer review. As part of this commitment we make the peer review history of every article we publish publicly available.

When an article is published we post the peer reviewers' comments and the authors' responses online. We also post the versions of the paper that were used during peer review. These are the versions that the peer review comments apply to.

The versions of the paper that follow are the versions that were submitted during the peer review process. They are not the versions of record or the final published versions. They should not be cited or distributed as the published version of this manuscript.

BMJ Open is an open access journal and the full, final, typeset and author-corrected version of record of the manuscript is available on our site with no access controls, subscription charges or pay-per-view fees (http://bmjopen.bmj.com).

If you have any questions on BMJ Open's open peer review process please email info.bmjopen@bmj.com

BMJ Open

# A scoping review protocol to map the evidence on interventions to prevent overweight and obesity in children

| | |
|---|---|
| Journal: | *BMJ Open* |
| Manuscript ID | bmjopen-2017-019311 |
| Article Type: | Protocol |
| Date Submitted by the Author: | 24-Aug-2017 |
| Complete List of Authors: | Bussiek , Peer-Benedikt Vincent ; London School of Economics and Political Science, Management<br>De Poli, Chiara; London School of Economics and Political Science, Management<br>Bevan, Gwyn; The London School of Economics and Pol, Management |
| Keywords: | Children, Overweight, Obesity, Prevention, Scoping review, PUBLIC HEALTH |
| | |

SCHOLARONE™
Manuscripts

# A scoping review protocol to map the evidence on interventions to prevent overweight and obesity in children

Peer-Benedikt Vincent Bussiek, Department of Management, London School of Economics and Political Science, London, UK

Chiara De Poli, Department of Management, London School of Economics and Political Science, London, UK*

Gwyn Bevan, Department of Management, London School of Economics and Political Science, London, UK

* Corresponding author

Ms Chiara De Poli

Houghton Street

London WC2A 2AE

Email c.de-poli@lse.ac.uk

Phone number 02079556749



For peer review only - http://bmjopen.bmj.com/site/about/guidelines.xhtml

**ABSTRACT**

**Introduction.** Obesity has become one of the biggest public health problems of the 21st century. Prevalence of obesity in children and adolescents has dramatically increased worldwide in the past decades, and this trend is expected to continue in the future. Obesity in childhood is a good predictor of obesity in adulthood, which is a common risk factor for a wide array of chronic diseases, poor quality of life and reduced life expectancy. Obesity is preventable and a vast but fragmented body of evidence on interventions to prevent obesity in children is now available. In this article we outline the protocol for a scoping review of reviews of the literature published on the subject.

**Methods and analysis.** We draw upon the five-staged Arksey and O'Malley's framework to guide the scoping review process. Following the definition of our research questions (stage 1), we define the eligibility criteria and search strategy that we intend to use (stage 2). The study selection process based on the eligibility criteria identified in the previous phase will follow (stage 3). A framework developed for this review will then inform the extraction and charting of data from the included reviews (stage 4) and results will be aggregated and summarized with criteria relevant for health professionals and policy makers (stage 5).

**Ethics and dissemination.** We anticipate the results of the scoping review to provide a comprehensive overview of the evidence base of interventions to prevent obesity in children and to highlight areas where evidence is controversial or missing. It will also provide key information to policy makers and health professionals interested in planning, funding and delivering evidence-based, effective interventions to prevent children obesity.

**Registration.** Not required.

**Keywords:** Children, Overweight, Obesity, Prevention, Scoping review, Public Health



For peer review only - http://bmjopen.bmj.com/site/about/guidelines.xhtml

**Strengths and limitations of this study**

- The present protocol is for a scoping review of published systematic reviews, as a pragmatic way of dealing with the large evidence base now available on obesity prevention in children.
- The present scoping review aims to use eleven electronic databases and tailored search strings which have been iteratively refined in order to increase the likelihood of retrieving as many relevant published article reviews as possible.
- Only article reviews published in English in scientific journals will be considered eligible for inclusion.
- A quality assessment of the article reviews included in the scoping review will not be performed, as this would be beyond the aim of a scoping review.

For peer review only

## BACKGROUND

Overweight and obesity are defined “as abnormal or excessive fat accumulation that may impair health”[1] and are caused by a substantial energy imbalance.

Most of the world's population live in countries where overweight and obesity kill more people than underweight.[1]

Childhood obesity has become a global problem. In 2015, about 110 million children and young adults (under 20 years of age) were estimated to be obese, equivalent to an overall prevalence of 5%.[2] Epidemiological data available show that the number of overweight or obese infants and young children (aged 0 to 5 years) increased from 32 million globally in 1990 to 42 million in 2013. If these trends will continue, the number of overweight or obese infants and young children globally is expected to spiral up to 70 million by 2025.[1]

Obesity in childhood can affect a child’s immediate health, as it is associated with a number of physical and psychological comorbidities (e.g. asthma, dental caries, attention-deficit hyperactivity disorder)[3,4]. It can also impair educational attainment and quality of life and can have substantial long term health consequences.[5] Obese children are likely to remain obese as adults, thus experiencing greater risk of poorer health outcomes.[6]

In 2014, more than 1.9 billion of people aged 18 years and above were overweight (39% of the adult population worldwide) and over 600 million (13%) were obese.[1] The worldwide prevalence of obesity has shown a continuous increase since 1980, with some significant regional variations.[2]

Excessive body weight in adults is an established risk factor for a wide array of poor physical and mental health outcomes and chronic diseases[7–9], such as hypertension, type 2 diabetes, cardiovascular diseases, chronic kidney disease, ischemic stroke, musculoskeletal disorders



For peer review only - http://bmjopen.bmj.com/site/about/guidelines.xhtml

and several types of cancer. A bidirectional association between psychological problems and obesity has also been clearly established.[10,11]

There are two reasons for focusing on preventing obesity in childhood rather than preventing or treating it in adulthood. First, although the prevalence of childhood obesity is estimated to be lower than the prevalence of adult obesity (5% against 13%), the rate of increase in childhood obesity in many countries is alarmingly greater than the rate of increase in adult obesity.[2] These trends are expected to continue if no radical actions to tackle the epidemic are implemented.

Secondly, overweight and obese children and teens are much more likely to become obese as adults (compared to normal BMI children), and it is more difficult for these adults to lose the excess weight once they become obese. Options for treating obesity, pharmacological and surgical, are nowadays available but are costly, hence cannot afforded at scale, and can have complications. For this reason, early prevention during childhood is better than attempts at cure later in life.[12]

Obesity is caused by a combination of exposure to an unhealthy environment and inadequate behavioural responses to that environment. Hence, interventions aimed at successfully preventing obesity ought to address the obesogenic environment to which individuals are exposed, e.g. by supporting healthy eating and offering opportunities for physical activity, as well as critical developmental, biological or behavioural factors over the life-course of individuals.[13]

Strategies against childhood obesity currently implemented in many countries aim to combine behavioural, lifestyle interventions targeting particular sub-populations and initiatives that benefit the wider population of children. For example, in England, lifestyle weight management services are available for overweight or obese children and young



For peer review only - http://bmjopen.bmj.com/site/about/guidelines.xhtml

people[14] alongside population-based interventions, such as a soft drinks industry levy, improved food labelling and a reduction of sugar in the products children eat most, which have been pledged in the Plan for Action against Childhood Obesity published by the Government in 2016.[15] Similar strategies are implemented in the US[16], Canada[17] and in many European countries.[18]

Obesity prevention in children is a complex problem. The obesity system map for the UK represents such complexity in a very powerful way. The map was developed as a heuristic tool to describe the anatomy of the obesity system and it includes more than 100 variables (clustered thematically around physiology, individual physical activity, the physical activity environment, food consumption, food production, individual psychology and social psychology) and several intricate loops representing causal linkages between the different variables.[19] Different disciplines and research approaches have been deployed over time producing a vast and diverse evidence base. In order to inform evidence based policies to prevent obesity in children, we aim to carry out a scoping review of reviews of the literature on interventions to prevent childhood obesity. The objective is to understand what these interventions are, if they are effective, why they succeed and, if they fail, why they do so. A scoping review is an appropriate methodology for reviewing large bodies of literature in order to generate an overview of research undertaken on a topic and determine the range of studies that are available, summarize research results and identify evidence gaps.[20]

In this article, we present the protocol that will inform our scoping review.



For peer review only - http://bmjopen.bmj.com/site/about/guidelines.xhtml

## METHODS

The scoping review is based on the Arksey and O'Malley's framework[21] and following developments proposed by Levac, Colquhoun, & O'Brien[22] and the Joanna Briggs Institute[23], which recommend to organize the review process in (at least) five stages[21]

- Stage 1. Identifying the research question
- Stage 2. Identifying relevant studies
- Stage 3. Study selection
- Stage 4. Charting the data
- Stage 5. Collating, summarising and reporting the results

### Stage 1. Identifying the research question

Preliminarily to identifying the research question, we initiated an iterative process to increase our familiarity with the literature on childhood obesity and subsequently refined the scope of our review. Throughout this process we decided not to use any criteria to restrict our review to specific study populations (e.g., specific age groups) as it became clear that overweight and obesity need to be addressed as early as possible and also opportunistically, as weight gain in children appears to be a strong predictor of obesity in adulthood.[24]

On the basis of the initial exploratory research, we identified the following research questions:

1. What types of interventions to prevent children's obesity are addressed in the literature?
2. What are the children populations targeted by these interventions?



For peer review only - http://bmjopen.bmj.com/site/about/guidelines.xhtml

3. In what settings are these interventions provided?

4. Are these interventions effective?

5. If these interventions are effective, what is the scale of the reduction in childhood obesity?

6. What are the barriers and facilitators to effective implementation of these interventions?

7. What evidence is there of the effectiveness of these interventions when they are combined?

For peer review only

**Stage 2. Identifying relevant studies**

Following the framework by Arksey and O'Malley's, in the second stage of the scoping review process we identified the criteria that will be used to select the studies for inclusion in the review. Although a scoping review is designed to cover a broad spectrum of literature, these criteria will guide our search and help filter for relevant sources.

The scoping review will include published systematic reviews that can be retrieved from the following electronic databases: Cochrane Database of Systematic Reviews, Cumulative Index to Nursing and Allied Health Literature (CINAHL), ERIC, Google Scholar, Joanna Briggs Library, MEDLine / PubMed, NHS EED, PsycInfo, Scopus, SocIndex and Web of Science. Reference lists of reviews found through the electronic search will be checked to ensure that relevant articles are included in the scoping review.

Based on the initial exploratory research, we agreed the following eligibility criteria:

– Type of publication: journal articles

– Time frame: any



For peer review only - http://bmjopen.bmj.com/site/about/guidelines.xhtml

- Language: English
- Study population: children and adolescents, aged below 18
- Types of intervention: interventions aiming at preventing childhood obesity
- Types of review articles: systematic reviews, meta-analyses, scoping reviews, evidence maps, rapid reviews, literature reviews, evidence syntheses, reviews of reviews, narrative reviews, critical reviews

Based on the initial scoping process, we decided to exclude conference abstracts, book reviews, commentaries or editorial articles, to exclude reviews focusing on individuals aged 18 or above and reviews focusing on the treatment of obesity (e.g. bariatric surgery), rather than on its prevention.

As suggested by Levac, Colquhoun, and O'Brien[22], the team used an iterative process to identify key search terms. We started by using the following keywords: child*, obes*, weight*, intervention, prevent*, review. The review articles retrieved were then screened for their titles, abstracts and index terms. We also consulted with an academic librarian who advised on the most appropriate MESH terms for our search and how to modify them for the different databases used. Based on this exploratory scoping phase, we finalized the search strings for each database (Supplementary material 1).

We ran the search and articles retrieved from each database were imported into a reference management software.

**Stage 3. Study selection**



For peer review only - http://bmjopen.bmj.com/site/about/guidelines.xhtml

The third stage of the Arksey and O'Malley's framework aims to identify the studies that will be included in the scoping review. A member of the team consolidated the results of the searches run on the different databases and removed studies retrieved from more than one database in order to exclude duplicates. He will screen titles and abstracts of the articles to exclude those which do not meet the eligibility criteria identified in the second stage of the protocol. For those fulfilling our eligibility criteria, we will retrieve the full article.

Another member of the team will screen a sample (i.e. 20%) of the retrieved articles to ensure a consistent application of the eligibility criteria for inclusion in the review. She will also review titles and abstracts of the articles for which the first reviewer could not determine whether they are eligible for inclusion. Disagreements about study eligibility of the sampled articles will be discussed between the two reviewers until consensus is reached or by arbitration of a third reviewer, if required. The process of study selection is reported using a PRISMA flow chart, which will be updated once the review is completed (Supplementary material 2).

**Stage 4. Charting the data**

Based on the preliminary scoping phase, we developed a data extraction framework with 18 categories that will be used to assess the full review articles retrieved from the literature fulfilling our eligibility criteria for inclusion (Table 1).



For peer review only - http://bmjopen.bmj.com/site/about/guidelines.xhtml

**Table 1 - Data extraction framework**

| Main category | Sub-category | Description |
|---|---|---|
| 1. Authors | | |
| 2. Title | | |
| 3. Journal | | |
| 4. Year of publication | | |
| 5. Objective(s) of the review | | Describe the stated objectives of the review |
| 6. Type of review | | Specify if systematic review and meta-analysis, scoping review, narrative review |
| 7. Number of studies included in the review | | Indicate the number of primary studies included in the review |
| 8. Years of publication of the studies included in the review | | Specify the range of years of publications of the studies included in the review |
| 9. Countries where the studies included in the review were conducted | | Specify the geographical areas covered by the studies included in the review |
| 10. Type of studies included in the review | | Specify if the review includes specific types of studies (e.g. RCTs, cost-effectiveness analyses, qualitative studies, modelling studies) |
| 11. Description of the intervention(s) | Type of intervention | Specify the type(s) of the interventions on which the review focuses (e.g. lifestyle interventions, weight management programmes, sugar taxes) |
| | Content of the intervention | Describe how, by whom, how often, for how long the intervention is delivered |
| 12. Definition of overweight/obesity | | Specify the definition of overweight/obesity used in the review |
| 13. Description of the study population | Target population | Specify if the intervention (i) targets individuals within sub-population groups or (ii) the broad population (e.g. in case of interventions on the built environment, national policies and regulation) |
| | By BMI (pre intervention) | Specify the distribution of the study population by BMI pre intervention |
| | By age | Specify the age groups covered by the review |
| | By sex | Specify if the review focuses on interventions targeting specifically boys or girls, or indicate the distribution of study participants by sex |
| | By ethnic background | Specify if the review focuses on interventions targeting specific ethnic groups (e.g. South Asians), or |



For peer review only - http://bmjopen.bmj.com/site/about/guidelines.xhtml

| Main category | Sub-category | Description |
|---|---|---|
| | | indicate the distribution of study participants by ethnic groups |
| | By socio-economic background | Specify if the review focuses on interventions targeting children living in deprived areas, or indicate the distribution of study participants by socio-economic background |
| | By other characteristics of the study population (e.g. disability, comorbidity) | Specify if the review focuses on specific populations (e.g. children with disabilities) |
| 14. Setting of the intervention(s) | | Specify if the review focuses on interventions delivered in school-, family-, community-based settings |
| 15. Reported outcomes | | Describe the intervention outcomes reported in the review (e.g. weight, BMI, self-efficacy) |
| 16. Effectiveness | | Describe the results reported in the review (e.g. change in BMI) |
| 17. Impact | | Describe the distribution of the study population by BMI (or other relevant outcomes) post intervention |
| 18. Facilitators | | Describe the factors that support or enable the implementation of the intervention reported in the review |
| 19. Barriers | | Describe the factors that inhibit the implementation of the intervention reported in the review |

For peer review only



For peer review only - http://bmjopen.bmj.com/site/about/guidelines.xhtml

Alongside standard bibliographic information (i.e. authors, title, journal, year of publication), type and objectives of the review will be reported. For each article, information on the interventions covered by the review, characteristics of the study populations, definition of overweight/obesity adopted in the reviews, settings of the interventions, types of outcomes assessed, information on the effectiveness of the interventions and facilitators and barriers to the implementation of the interventions will be tabled.

The framework will be pilot tested by two team members on a sample of the included studies (i.e. 10% of the complete list of retrieved studies) in order to ensure that the coding framework is consistently applied. If necessary, the categories will be modified and the data extraction framework revised accordingly. Questions arising when piloting the framework will be discussed by the team and possible disagreement will be resolved through team consultations.

The same two members of the team will be in charge of independently charting the data from each included review study, following the data extraction framework. In order to ensure inter-rater reliability, a sample (i.e. 20%) of the included articles independently reviewed will then be compared by the two members of the team. Discrepancies in extracted data will be discussed between the two reviewers until consensus is reached or by arbitration of a third reviewer, if required.

For peer review only

**Stage 5. Collating, summarising and reporting the results**

The analysis of the data collected using the data extraction framework will provide information on the body of research that has been conducted on interventions to prevent obesity in children. For example, evidence on the interventions to tackle children obesity will be presented by age groups in order to show at what point in the life-course what types of



For peer review only - http://bmjopen.bmj.com/site/about/guidelines.xhtml

interventions are more effective and, hence, worth pursuing. The analysis will highlight the clinical effectiveness of the interventions reviewed (e.g. the change in the BMI of participants included in the review studies) and their material impact on the population in need (e.g. how the distribution of study participants by BMI groups change before and after the intervention). It will also highlight if contrasting results have been reported for similar interventions and identify areas which have been under-researched and may require further investigation.

Results will be presented in an aggregate and visual form (e.g. using tables and charts), as appropriate.

## CONCLUSION

Childhood obesity is one of the greatest public health challenges of the 21$^{st}$ century and the current prevalence rates seem to suggest that progress in tackling the obesity epidemic has been so far slow and limited. In contrast with this failure to intervene effectively, the body of research on preventing obesity has increased exponentially and reviews of primary studies have been increasingly carried out to synthetize the evidence base now available. Given the quantity and variety of review studies published, a scoping review of the literature is a promising approach to systematize this growing evidence base, make it accessible to different knowledge users for different purposes and to surface knowledge gaps.

## REFERENCES

1. World Health Organization. Obesity and overweight - Fact sheet [Internet]. World Health Organization. World Health Organization; 2016. Available from: http://www.who.int/mediacentre/factsheets/fs311/en/



For peer review only - http://bmjopen.bmj.com/site/about/guidelines.xhtml

2. The GBD 2015 Obesity Collaborators. Health Effects of Overweight and Obesity in 195 Countries over 25 Years. N Engl J Med. 2017;NEJMoa1614362.

3. Pulgarón ER, Shor B, Tanaka CY, Hayes DK. Childhood obesity: a review of increased risk for physical and psychological comorbidities. Clin Ther. 2013;35(1):A18-32.

4. Kalarchian MA, Marcus MD. Psychiatric comorbidity of childhood obesity. Int Rev Psychiatry. 2012;24(3):241–6.

5. Hawkes C, Popkin BM. Can the sustainable development goals reduce the burden of nutrition-related non-communicable diseases without truly addressing major food system reforms? BMC Med. 2015;13:143.

6. Juonala M, Magnussen CG, Berenson GS, Venn A, Burns TL, Sabin MA, et al. Childhood Adiposity, Adult Adiposity, and Cardiovascular Risk Factors. N Engl J Med. 2011;365(20):1876–85.

7. Field AE, Coakley EH, Must A, Spadano JL, Laird N, Dietz WH, et al. Impact of Overweight on the Risk of Developing Common Chronic Diseases During a 10-Year Period. Arch Intern Med. 2001;161(13):1581.

8. Padula W V., Allen RR, Nair K V. Determining the cost of obesity and its common comorbidities from a commercial claims database. Clin Obes. 2014;4(1):53–8.

9. Haslam DW, James WPT. Obesity. Lancet. 2005 Oct;366(9492):1197–209.

10. Luppino FS, de Wit LM, Bouvy PF, Stijnen T, Cuijpers P, Penninx BWJH, et al. Overweight, Obesity, and Depression. Arch Gen Psychiatry. 2010;67(3):220.

11. Lin H-Y, Huang C-K, Tai C-M, Lin H-Y, Kao Y-H, Tsai C-C, et al. Psychiatric disorders of patients seeking obesity treatment. BMC Psychiatry. 2013;13(1):1.



For peer review only - http://bmjopen.bmj.com/site/about/guidelines.xhtml

12. Pandita A, Sharma D, Pandita D, Pawar S, Tariq M, Kaul A. Childhood obesity: prevention is better than cure. Diabetes Metab Syndr Obes. 2016;9:83–9.

13. WHO Commission on Ending Childhood Obesity. Ending Childhood Obesity. Geneva; 2016.

14. National Institute for Health and Care Excellence (NICE). Lifestyle weight management services for overweight or obese children and young people overview - NICE Pathways [Internet]. Available from: https://pathways.nice.org.uk/pathways/lifestyle-weight-management-services-for-overweight-or-obese-children-and-young-people#content=view-node%3Anodes-developing-a-tailored-plan

15. HM Government. Childhood Obesity A Plan for Action. London, UK; 2016.

16. Centers for Disease Control and Prevention (CDC). Nutrition, Physical Activity, and Obesity Prevention Strategies [Internet]. Prevention Strategies & Guidelines. Available from: https://www.cdc.gov/obesity/resources/strategies-guidelines.html

17. Government of Canada. Curbing Childhood Obesity A Federal, Provincial and Territorial Framework for Action to Promote Healthy Weights. Government of Canada; 2010.

18. European Commission - High Level Group on Nutrition and Physical activity. EU Action Plan on Childhood Obesity 2014-2020. Brussels, Belgium: European Commission; 2014.

19. Vandenbroeck IP, Goossens J, Clemens M. Foresight Tackling Obesities: Future Choices — Obesity System Atlas. London, UK; 2007.

20. Wickremasinghe D, Kuruvilla S, Mays N, Avan BI. Taking knowledge users' knowledge needs into account in health: an evidence synthesis framework. Health



For peer review only

For peer review only - http://bmjopen.bmj.com/site/about/guidelines.xhtml

Policy Plan. 2016 May;31(4):527–37.

21. Arksey H, O'Malley L. Scoping studies: towards a methodological framework. Int J Soc Res Methodol. 2005 Feb;8(1):19–32.

22. Levac D, Colquhoun H, O'Brien KK. Scoping studies: advancing the methodology. Implement Sci. 2010;5:69.

23. The Joanna Briggs Institute. The Joanna Briggs Institute Reviewers' Manual 2015 - Methodology for JBI Scoping Reviews. Adelaide, South Australia; 2015.

24. Pearce M, Webb-Phillips S, Bray I. Changes in objectively measured BMI in children aged 4-11 years: Data from the National Child Measurement Programme. J Public Heal (United Kingdom). 2016;38(3):459–66.

**FUNDING STATEMENT**

PBVB and GB were funded by the Department of Management at the London School of Economics and Political Science. CDP was funded by the National Institute for Health Research Collaboration for Leadership in Applied Health Research and Care (NIHR CLAHRC) North Thames at Barts Health NHS Trust. The views expressed are those of the authors and not necessarily those of the NHS, the NIHR or the Department of Health.

**COMPETING INTERESTS STATEMENT**

None declared

**AUTHORS' CONTRIBUTIONS**



For peer review only - http://bmjopen.bmj.com/site/about/guidelines.xhtml

PBVB contributed to develop the research questions, the methods (searching the databases, managing the retrieved records, removing duplicates) and to draft and edit the protocol. CDP conceived of the idea the scoping review, developed the research questions and contributed to the development of the methods. She contributed extensively to draft and edit the manuscript. GB supervised the preparation of the protocol and critically reviewed the manuscript. All Authors have approved the final manuscript.

## DATA SHARING STATEMENT

No additional data are available.

For peer review only



For peer review only - http://bmjopen.bmj.com/site/about/guidelines.xhtml

# Supplementary material 1

## Search strategy

### CINAHL Plus via EBSCOhost

| | |
|---|---|
| **Date of search** | 18/08/2017 |
| **Search string** | (TI ( obesity OR overweight OR weight ) OR MH (obesity OR pediatric obesity)) AND (TI ( scoping OR systematic OR meta OR review ) OR MH (review OR meta-analysis)) AND (AB ( child* OR adolescen* OR you* ) OR MH (child OR adolescent)) AND AB ( prevention OR intervention ) |
| **Number of results** | 178 for AB; 185 for SU |
| **Number of results** | 363 |

### Cochrane Library of Systematic Reviews

| | |
|---|---|
| **Date of search** | 18/08/2017 |
| **Search string** | MeSH descriptor: [Pediatric Obesity] explode all trees |
| **Number of results** | 12 |

### ERIC

| | |
|---|---|
| **Date of search** | 26/07/2017 |
| **Search string** | (title:(obesity OR overweight OR weight) AND (title:(scoping OR systematic OR meta OR review)) AND (abstract:(child* OR adolescen* OR you*)) |
| **Number of results** | 454 |

### ERIC via EBSCOhost

| | |
|---|---|
| **Date of search** | 26/07/2017 |
| **Search string** | TI ( obesity OR overweight OR weight ) AND TI ( scoping OR systematic OR meta OR review ) AND AB ( child* OR adolescen* OR you* ) AND AB ( prevention OR intervention ) |
| **Number of results** | 35 |

### Google Scholar

| | |
|---|---|
| **Date of search** | 26/07/2017 |
| **Search string** | (obesity OR overweight OR weight) (scoping OR systematic OR meta OR review) ( child OR adolescen OR you ) (prevention OR intervention)<br>allintitle: (obesity OR overweight OR weight) (scoping OR systematic OR meta OR review) (child OR adolescen OR you) |
| **Number of results** | 104 |



For peer review only - http://bmjopen.bmj.com/site/about/guidelines.xhtml

**Joanna Briggs Institute (JBI) Database of Systematic Reviews and Implementation Reports via OVID**

| | |
|---|---|
| **Date of search** | 28/07/2017 |
| **Search string** | (Ti: ((obesity OR overweight OR weight) AND (scoping OR systematic OR meta OR review))) AND (ab:((child* OR adolescen* OR you*) AND (prevention OR intervention)) |
| **Number of results** | 31 |

**MEDLine/PubdMed MeSH**

| | |
|---|---|
| **Date of search** | 18/08/2017 |
| **Search string** | ((((obesity[Title] OR overweight[Title] OR weight[Title] OR obesity[MeSH Terms] OR pediatric obesity[MeSH Terms])) AND (scoping[Title] OR systematic[Title] OR meta[Title] OR review[Title] OR Review[MeSH Terms] OR Meta-analysis[MeSH-Terms])) AND (child*[Title/Abstract] OR adolescen*[Title/Abstract] OR you*[Title/Abstract] OR Child[MeSH Terms] OR Adolescent[MeSH Terms])) AND (prevention[Title/Abstract] OR intervention[Title/Abstract]) |
| **Number of results** | 792 |

**NHS EED**

| | |
|---|---|
| **Date of search** | 28/07/2017 |
| **Search string** | ((obesity OR overweight OR weight)):TI OR ((scoping OR systematic OR meta OR review)):TI OR ((child* OR adolescen* OR you*)):TI AND ((prevention OR intervention)):TI |
| **Number of results** | 99 |

**PsycINFO via EBSCOhost**

| | |
|---|---|
| **Date of search** | 28/07/2017 |
| **Search string** | TI ( obesity OR overweight OR weight ) AND TI ( scoping OR systematic OR meta OR review ) AND AB ( child* OR adolescen* OR you* ) AND AB ( prevention OR intervention ) |
| **Number of results** | 275 |

**Scopus**

| | |
|---|---|
| **Date of search** | 28/07/2017 |
| **Search string** | TITLE( obesity OR overweight OR weight ) AND TITLE( scoping OR systematic OR meta OR review ) AND ABS( child* OR adolescen* OR you* ) AND ABS( prevention OR intervention ) |



For peer review only - http://bmjopen.bmj.com/site/about/guidelines.xhtml

| | |
|---|---|
| **Number of results** | 501 |

**SocINDEX via EBSCOhost**

| | |
|---|---|
| **Date of search** | 28/07/2017 |
| **Search string** | TI ( obesity OR overweight OR weight ) AND TI ( scoping OR systematic OR meta OR review ) AND AB ( child* OR adolescen* OR you* ) AND AB ( prevention OR intervention ) |
| **Number of results** | 20 |

**Web of Science / Web of Knowledge**

| | |
|---|---|
| **Date of search** | 18/07/2017 |
| **Search string** | TI=((obesity OR overweight OR weight) AND (scoping OR systematic OR meta OR review)) AND TS=((child* OR adolescen* OR you*) AND (prevention OR intervention)) |
| **Number of results** | 567 |

For peer review only

# Supplementary material 2

## PRIMA flowchart

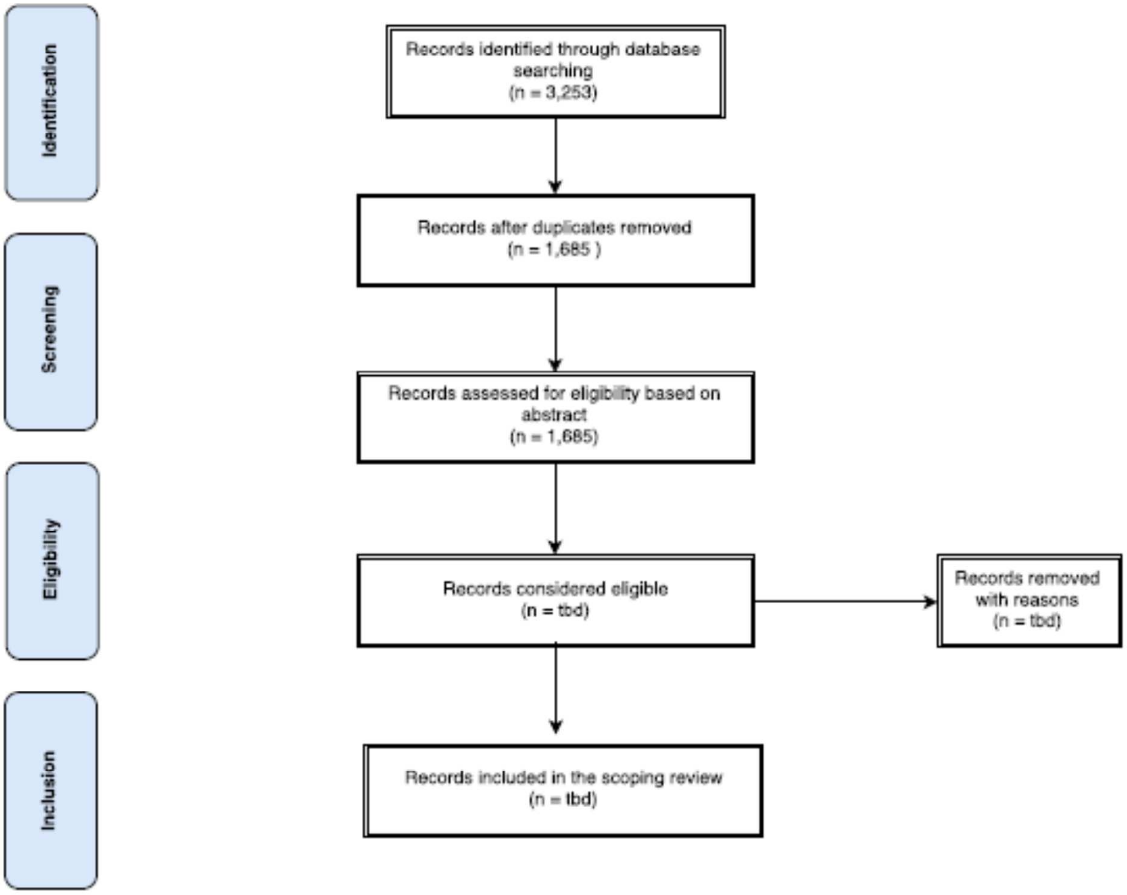

BMJ Open

# A scoping review protocol to map the evidence on interventions to prevent overweight and obesity in children

| | |
|---|---|
| Journal: | *BMJ Open* |
| Manuscript ID | bmjopen-2017-019311.R1 |
| Article Type: | Protocol |
| Date Submitted by the Author: | 03-Jan-2018 |
| Complete List of Authors: | Bussiek , Peer-Benedikt Vincent ; London School of Economics and Political Science, Management<br>De Poli, Chiara; London School of Economics and Political Science, Management<br>Bevan, Gwyn; The London School of Economics and Pol, Management |
| <b>Primary Subject Heading</b>: | Public health |
| Secondary Subject Heading: | Public health |
| Keywords: | Children, Overweight, Obesity, Prevention, Scoping review |
| | |

SCHOLARONE™ Manuscripts

For peer review only

# A scoping review protocol to map the evidence on interventions to prevent overweight and obesity in children

Peer-Benedikt Vincent Bussiek, Department of Management, London School of Economics and Political Science, London, UK

Chiara De Poli, Department of Management, London School of Economics and Political Science, London, UK*

Gwyn Bevan, Department of Management, London School of Economics and Political Science, London, UK

* Corresponding author

Ms Chiara De Poli

Houghton Street

London WC2A 2AE

Email c.de-poli@lse.ac.uk

Phone number 02079556749

For peer review only



For peer review only - http://bmjopen.bmj.com/site/about/guidelines.xhtml

## ABSTRACT

**Introduction.** Obesity has become one of the biggest public health problems of the 21st century. Prevalence of obesity in children and adolescents has increased dramatically worldwide over the last 20 years, and this trend is expected to continue. Obesity in childhood is concerning as it predicts obesity in adulthood, a common risk factors for a wide array of chronic diseases and poor health outcomes. Obesity is preventable and a vast but fragmented body of evidence on preventative interventions is now available. This article outlines the protocol for a scoping review of published literature reviews on interventions to prevent obesity in children. The scoping review addresses the broad research question What is the evidence on interventions to prevent childhood obesity? and aims to give an overview of the various interventions available, understand those which are effective and identify barriers and facilitators to their effectiveness.

**Methods and analysis.** The six-staged Arksey and O'Malley methodology framework is used to guide the scoping review process. Following the definition of the research questions (stage 1), the eligibility criteria and search strategy are defined (stage 2). The study selection process based on the eligibility criteria identified will follow (stage 3). A framework developed for this review will then inform the extraction and charting of data from the included reviews (stage 4) and results will be aggregated and summarized with criteria relevant for health professionals and policy makers (stage 5). The optional consultation (stage 6) exercise is not planned.

**Ethics and dissemination.** Since the scoping review methodology aims at synthetizing information from available publications, this study does not require ethical approval. An article reporting the results of the scoping review will be submitted for publication to a scientific journal, presented at relevant conferences and disseminated as part of future workshops with professionals involved in obesity prevention.



For peer review only - http://bmjopen.bmj.com/site/about/guidelines.xhtml

For peer review only

**Registration.** Not required.

**Keywords:** Children, Overweight, Obesity, Prevention, Scoping review, Public Health

**Strengths and limitations of this study**

- The present protocol is for a scoping review of published systematic reviews, as a pragmatic way of dealing with the large evidence base available on obesity prevention in children
- The present scoping review aims to use eleven electronic databases and tailored search strings which have been iteratively refined in order to increase the likelihood of retrieving as many relevant published article reviews as possible
- Only article reviews published in English in scientific journals will be considered eligible for inclusion
- A quality assessment of the article reviews included in the scoping review will not be performed, as this would be beyond the aim of a scoping review

For peer review only

## BACKGROUND

Childhood obesity, the abnormal or excessive fat accumulation that may impair health,[1] has become a global problem. In 2015, about 110 million children and young adults (under 20 years of age) were estimated to be obese, equivalent to an overall prevalence of 5%.[2] Epidemiological data show that the number of overweight or obese infants and young children (aged 0 to 5 years) increased from 32 million globally in 1990 to 42 million in 2013.



For peer review only - http://bmjopen.bmj.com/site/about/guidelines.xhtml

If these trends will continue, the number of overweight or obese infants and young children is expected to spiral up to 70 million by 2025.[1]

Although the prevalence of childhood obesity is estimated to be lower than the prevalence of adult obesity (5% against 13%), the rate of increase in childhood obesity in many countries is alarmingly greater than the rate of increase in adult obesity.[2] These trends are expected to continue if no radical actions to tackle the epidemic are implemented.

Obesity in childhood can affect a child's immediate health, as it is associated with a number of physical and psychological comorbidities (e.g. asthma, dental caries, attention-deficit hyperactivity disorder, non-alcoholic fatty liver disease).[3–6] It can also impair educational attainment[7–9] and quality of life[10,11] and can have substantial long term health consequences into adulthood.[12] There is evidence that an early adiposity rebound, the point in life when BMI rises again after reaching a nadir, predicts later obesity.[13–15] Hence, obese children are more likely than children with normal BMI to remain obese as adults[12,16–20] and to experience greater risk of poorer health outcomes.[21,22]

Options for treating obesity, pharmacological and surgical, are nowadays available but are costly, hence cannot afforded at scale, and can have complications. For these reasons, early prevention during childhood is better than attempts at cure later in life.[23]

However, obesity prevention in children is a complex task. The obesity system map for the UK represents such complexity in a powerful way. The map was developed as a heuristic tool to describe the anatomy of the obesity system and it includes more than 100 variables (clustered thematically around physiology, individual physical activity, the physical activity environment, food consumption, food production, individual psychology and social psychology) and several intricate loops representing causal linkages between the different variables.[24] To confront this complexity, strategies to tackle childhood obesity that are



For peer review only - http://bmjopen.bmj.com/site/about/guidelines.xhtml

currently implemented in many countries combine behavioural, lifestyle interventions targeting particular sub-populations and initiatives addressing the obesogenic environment to which the wider population is exposed.[25] As such they intend to address the energy imbalance that leads to obesity, caused by a combination of exposure to an unhealthy environment as well as inadequate behavioural responses to that environment and the specific needs of a person.[26–28]

For example, in England, lifestyle weight management services are available for overweight or obese children and young people[29] alongside population-based interventions, such as a soft drinks industry levy, improved food labelling and a reduction of sugar in the products children eat most, which have been pledged in the Plan for Action against Childhood Obesity published by the UK Government in 2016.[30] Similar strategies are implemented in the US,[31] Canada[32] and in many European countries.[33]

In order to inform evidence-based policies in the area of obesity prevention in children, a synthesis of the body of evidence currently available is needed.[13,34] To this aim, a scoping review of reviews of the literature on interventions to prevent childhood obesity is proposed with the objective of providing a descriptive overview of what these interventions are, if they are effective and why they succeed (or do not).

The scoping review represents an appropriate methodology for reviewing large bodies of literature in order to generate an overview of research undertaken on a topic and determine the range of studies that are available, summarize research results and identify evidence gaps.[35] As such, they do not aim at critically appraising individual studies which may be in fact heterogenous in terms of study design, methodology and, hence, quality of the results reported.[36] Despite this limitation, a scoping review of the literature on the topic identified will be valuable for at least two reasons. Firstly, the present scoping review aims to overcome the narrow foci of the few scoping reviews already available on prevention of childhood



For peer review only - http://bmjopen.bmj.com/site/about/guidelines.xhtml

obesity (e.g. interventions delivered in specific settings, such as schools)[37–40] and to adopt a comprehensive approach to the topic. Secondly, over the last ten years, several systematic reviews have been published (see for example [41–44]), and a synthesis of this growing evidence base is now due. In this article, the protocol that will inform the scoping review is presented.

## METHODS AND ANALYSIS

### Protocol design

The scoping review is informed by the framework proposed by Arksey and O'Malley[36] which has been further developed by Levac, Colquhoun, & O'Brien[45] and the Joanna Briggs Institute.[46] This recommends to organize the review process in at least five stages[36]

– Stage 1. Identifying the research question
– Stage 2. Identifying relevant studies
– Stage 3. Study selection
– Stage 4. Charting the data
– Stage 5. Collating, summarising and reporting the results

The original framework proposed by Arksey and O'Malley suggests an optional consultation exercise (stage 6) with key stakeholders in order to identify additional references about potential studies to include as well as collect their feedback about the findings uncovered by the scoping review. Although a consultation with key stakeholders would represent a valuable exercise, the present scoping review will not encompass one because of time and budget constraints.



For peer review only - http://bmjopen.bmj.com/site/about/guidelines.xhtml

**Stage 1. Identifying the research questions**

Preliminarily to identifying the research question, an exploratory review of the literature on childhood obesity helped refine the scope of the present protocol. This phase informed the decision not to use any criteria to restrict the review to specific study populations (e.g., specific age groups) as it became clear that overweight and obesity need to be addressed as early as possible and also opportunistically, as weight gain in children appears to be a good predictor of obesity in adulthood.[12,16–20]

On the basis of the initial exploratory research, the following research questions were identified:

1. What types of interventions to prevent children's obesity are addressed in the literature?
2. What are the children populations targeted by these interventions?
3. In what settings are these interventions provided?
4. Are these interventions effective?
5. Which measures are used to assess obesity in children?
6. If these interventions are effective, what is the scale of the reduction in childhood obesity?
7. What are the barriers and facilitators to effective implementation of these interventions?
8. What evidence is there of the effectiveness of these interventions when they are combined?

**Stage 2. Identifying relevant studies**



For peer review only - http://bmjopen.bmj.com/site/about/guidelines.xhtml

For peer review only

Following the framework of Arksey and O'Malley, the second stage of the scoping review process aimed to identify the criteria that will be used to select the studies for inclusion in the review. Although a scoping review is designed to cover a broad spectrum of literature, these criteria will guide the search and help filter for relevant sources.

The scoping review will include published systematic reviews that can be retrieved from the following electronic databases: Cochrane Database of Systematic Reviews, Cumulative Index to Nursing and Allied Health Literature (CINAHL), ERIC, Google Scholar, Joanna Briggs Library, MEDLine / PubMed, NHS EED, PsycInfo, Scopus, SocIndex and Web of Science. Reference lists of reviews found through the electronic search will be checked to ensure that relevant articles are included in the scoping review.

Based on the initial exploratory research, we agreed the following eligibility criteria:

- Type of publication: journal articles
- Time frame: any
- Language: English
- Study population: children and adolescents, aged below 18
- Types of intervention: interventions aiming at preventing childhood obesity
- Types of review articles: systematic reviews, meta-analyses, scoping reviews, evidence map, rapid reviews, literature reviews, evidence syntheses, reviews of reviews, narrative reviews, critical reviews

Based on the initial scoping process, it was agreed to exclude: conference abstracts, book reviews, commentaries or editorial articles, reviews focusing on the adult population



For peer review only - http://bmjopen.bmj.com/site/about/guidelines.xhtml

(individuals aged 18 year or above) and reviews focusing on interventions for the treatment of obesity (e.g. bariatric surgery), rather than its prevention.

As suggested by Levac, Colquhoun, and O'Brien,[45] the team used an iterative process to identify also key search terms. Initially, the following keywords were used: child*, obes*, weight*, intervention, prevent*, review. The review articles retrieved were then screened for their titles, abstracts and index terms. An academic librarian was consulted and advised on the most appropriate MESH terms for the search and how to modify them for the different databases used. Based on this exploratory scoping phase, the search strings for each database were finalized (Supplementary material 1). Articles were retrieved from each database and imported into a reference management software.

**Stage 3. Study selection**

The third stage of the framework of Arksey and O'Malley's framework aims to identify the studies that will be included in the scoping review. The team consolidated the results of the searches run on the different databases and removed studies retrieved from more than one database in order to exclude duplicates. A member of the team will then screen titles and abstracts of the articles to exclude those that do not meet the eligibility criteria identified in the second stage of the protocol. For those fulfilling the eligibility criteria, the full article will be retrieved.

A sample (i.e. 20%) of the retrieved articles will be screened by another team member to ensure a consistent application of the eligibility criteria for inclusion in the review. Titles and abstracts of the articles for which the first reviewer could not determine whether they are eligible for inclusion will also be reviewed. Disagreements about study eligibility of the sampled articles will be discussed between the two reviewers until consensus is reached or by



For peer review only - http://bmjopen.bmj.com/site/about/guidelines.xhtml

arbitration of a third reviewer, if required. The process of study selection is reported using a PRISMA flow chart, which will be updated once the review is completed (Supplementary material 2).

**Stage 4. Charting the data**

Based on the preliminary scoping phase, a data extraction framework was developed. It includes 19 categories that will be used to assess the full review articles retrieved from the literature fulfilling the eligibility criteria for inclusion (Table 1).

For peer review only



For peer review only - http://bmjopen.bmj.com/site/about/guidelines.xhtml

**Table 1 - Data extraction framework**

| Main category | Sub-category | Description |
|---|---|---|
| 1. Authors | | |
| 2. Title | | |
| 3. Journal | | |
| 4. Year of publication | | |
| 5. Objective(s) of the review | | Describe the stated objectives of the review |
| 6. Type of review | | Specify if systematic review and meta-analysis, scoping review, narrative review |
| 7. Number of studies included in the review | | Indicate the number of primary studies included in the review |
| 8. Years of publication of the studies included in the review | | Specify the range of the years of publications of the studies included in the review |
| 9. Countries where the studies included in the review were conducted | | Specify the geographical areas covered by the studies included in the review |
| 10. Type of studies included in the review | | Specify if the review includes specific types of studies (e.g. RCTs, cost-effectiveness analyses, qualitative studies, modelling studies) |
| 11. Description of the intervention(s) | Type of intervention | Specify the type(s) of the interventions on which the review focuses (e.g. lifestyle interventions, weight management programmes, sugar taxes) |
| | Content of the intervention | Describe how and by whom the intervention is delivered |
| | Length and intensity of the intervention | Describe for how long the intervention is delivered and its intensity |
| 12. Definition of overweight/obesity | | Specify the definition of overweight/obesity used in the review |
| 13. Description of the study population | Target population | Specify if the intervention (i) targets individuals within sub-population groups or (ii) the broad population (e.g. in case of interventions on the built environment, national policies and regulation) |
| | By BMI (pre intervention) | Specify the distribution of the study population by BMI pre intervention |
| | By age | Specify the age groups covered by the review |
| | By sex | Specify if the review focuses on interventions targeting specifically boys or girls, or indicate the distribution of study participants by sex |



For peer review only - http://bmjopen.bmj.com/site/about/guidelines.xhtml

| Main category | Sub-category | Description |
|---|---|---|
| | By ethnic background | Specify if the review focuses on interventions targeting specific ethnic groups (e.g. South Asians), or indicate the distribution of study participants by ethnic groups |
| | By socio-economic background | Specify if the review focuses on interventions targeting children living in deprived areas, or indicate the distribution of study participants by socio-economic background |
| | By other characteristics of the study population (e.g. disability, comorbidity) | Specify if the review focuses on specific populations (e.g. children with disabilities) |
| 14. Setting of the intervention(s) | | Specify if the review focuses on interventions delivered in school-, family-, community-based settings |
| 15. Reported outcomes | | Describe the intervention outcomes reported in the review (e.g. weight, BMI, self-efficacy) |
| 16. Effectiveness | | Describe the results reported in the review (e.g. change in BMI) |
| 17. Impact | | Describe the distribution of the study population by BMI (or other relevant outcomes) post intervention |
| 18. Facilitators | | Describe the factors that support or enable the implementation of the intervention reported in the review |
| 19. Barriers | | Describe the factors that inhibit the implementation of the intervention reported in the review |



For peer review only - http://bmjopen.bmj.com/site/about/guidelines.xhtml

Alongside standard bibliographic information (i.e. authors, title, journal, year of publication), type and objectives of the review will be reported. For each article, information on the interventions covered by the review, characteristics of the study populations, definition of overweight/obesity adopted in the reviews, setting, length and intensity of the interventions, types of outcomes assessed, information on the effectiveness of the interventions and facilitators and barriers to the implementation of the interventions will be tabled.

The framework will be pilot tested by two team members on a sample of the included studies (i.e. 10% of the complete list of retrieved studies) in order to ensure that the coding framework is consistently applied. If necessary, the categories will be modified and the data extraction framework revised accordingly. Questions arising when piloting the framework will be discussed by the team and possible disagreement will be resolved through team consultations.

The same two members of the team will be in charge of independently charting the data from each included review study, following the data extraction framework. In order to ensure inter-rater reliability, a sample (i.e. 20%) of the included articles independently reviewed will then be compared by the two members of the team. Discrepancies in extracted data will be discussed between the two reviewers until consensus is reached or by arbitration of a third reviewer, if required.

**Stage 5. Collating, summarising and reporting the results**

The analysis of the data collected using the data extraction framework will provide information on the body of research that has been conducted on interventions to prevent obesity in children. For example, evidence on the interventions to tackle children obesity will be presented by age groups in order to show at what point in the life-course what types of



For peer review only - http://bmjopen.bmj.com/site/about/guidelines.xhtml

For peer review only

interventions are more effective and, hence, worth pursuing. Also, it will be possible to highlight the clinical effectiveness of the interventions reviewed (e.g. the change in the BMI of participants included in the review studies) and their material impact on the population in need (e.g. how the distribution of study participants by BMI groups changes before and after the intervention). Conversely, it will also show areas that have been under-researched and may require further investigation. Results will be presented in an aggregate and visual form (e.g. using tables and charts), as appropriate.

## ETHICS AND DISSEMINATION

Since the scoping review methodology aims at synthetizing information from publicly available publications, this study does not require ethical approval. In terms of dissemination activities, an article reporting the results of the scoping review will be submitted for publication to a scientific journal and presented at relevant conferences. We anticipate the results of the scoping review to provide a comprehensive overview of the evidence base of interventions to prevent obesity in children and to highlight areas where evidence is controversial or missing. It will also provide key information to policy makers and health professionals interested in planning, funding and delivering evidence-based, effective interventions to prevent children obesity. For this reason, the results will be also disseminated as part of future workshops with professionals involved in obesity prevention.

## REFERENCES

1. World Health Organization. Obesity and overweight - Fact sheet [Internet]. World Health Organization. World Health Organization; 2016. Available from: http://www.who.int/mediacentre/factsheets/fs311/en/



For peer review only - http://bmjopen.bmj.com/site/about/guidelines.xhtml

2. The GBD 2015 Obesity Collaborators. Health Effects of Overweight and Obesity in 195 Countries over 25 Years. N Engl J Med. 2017;NEJMoa1614362.

3. Barshop NJ, Francis CS, Schwimmer JB, Lavine JE. Nonalcoholic fatty liver disease as a comorbidity of childhood obesity.

4. Félix DR, Costenaro F, Bertaso C, Gottschall A, Perdomo Coral G. Non-alcoholic fatty liver disease (NAFLD) in obese children-effect of refined carbohydrates in diet.

5. Pulgarón ER, Shor B, Tanaka CY, Hayes DK. Childhood obesity: a review of increased risk for physical and psychological comorbidities. Clin Ther. 2013;35(1):A18-32.

6. Kalarchian MA, Marcus MD. Psychiatric comorbidity of childhood obesity. Int Rev Psychiatry. 2012;24(3):241–6.

7. Cohen AK, Rai M, Rehkopf DH, Abrams B. Educational attainment and obesity: a systematic review. Obes Rev. 2013 Dec;14(12):989–1005.

8. Miller AL. Neurocognitive Processes and Pediatric Obesity Interventions. Pediatr Clin North Am. 2016 Jun;63(3):447–57.

9. Pizzi MA, Vroman K. Childhood Obesity: Effects on Children's Participation, Mental Health, and Psychosocial Development. Occup Ther Heal Care. 2013 Apr 19;27(2):99–112.

10. Buttitta M, Iliescu C, Rousseau A, Guerrien A. Quality of life in overweight and obese children and adolescents: a literature review. Qual LIFE Res. 2014 May;23(4):1117–39.

11. Tsiros MD, Olds T, Buckley JD, Grimshaw P, Brennan L, Walkley J, et al. Health-related quality of life in obese children and adolescents. Int J Obes. 2009 Apr



For peer review only - http://bmjopen.bmj.com/site/about/guidelines.xhtml

3;33(4):387–400.

12. Kelsey MM, Zaepfel A, Bjornstad P, Nadeau KJ. Age-Related Consequences of Childhood Obesity. Gerontology. 2014;60(3):222–8.

13. Wofford LG. Systematic review of childhood obesity prevention. J Pediatr Nurs. 2008 Feb;23(1):5–19.

14. van Hoek E, Feskens EJM, Bouwman LI, Janse AJ. Effective interventions in overweight or obese young children: systematic review and meta-analysis. Child Obes. 2014 Dec;10(6):448–60.

15. Pearce M, Webb-Phillips S, Bray I. Changes in objectively measured BMI in children aged 4-11 years: Data from the National Child Measurement Programme. J Public Heal (United Kingdom). 2016;38(3):459–66.

16. Middleton P, Crowther CA, Simmonds L. Different intensities of glycaemic control for pregnant women with pre-existing diabetes. In: Middleton P, editor. Cochrane Database of Systematic Reviews. Chichester, UK: John Wiley & Sons, Ltd; 2016.

17. Whitaker DJ, Le B, Hanson RK, Baker CK, McMahon PM, Ryan G, et al. Risk Factors for the Perpetration of Child Sexual Abuse: A Review and Meta-Analysis. Vol. 32, Child Abuse & Neglect: The International Journal. p. 529–48.

18. Juonala M, Kelly R, Magnussen CG, Sabin MA, Cheung M. Development of hypertension in overweight adolescents: a review. Adolesc Health Med Ther. 2015 Oct;171.

19. Deshmukh-Taskar P, Nicklas TA, Morales M, Yang S-J, Zakeri I, Berenson GS. Tracking of overweight status from childhood to young adulthood: the Bogalusa Heart Study. Eur J Clin Nutr. 2006 Jan 31;60(1):48–57.

For peer review only

20. Guo SS, Chumlea WC. Tracking of body mass index in children in relation to overweight in adulthood. Am J Clin Nutr. 1999 Jul;70(1):145S–8S.

21. Juonala M, Magnussen CG, Berenson GS, Venn A, Burns TL, Sabin MA, et al. Childhood Adiposity, Adult Adiposity, and Cardiovascular Risk Factors. N Engl J Med. 2011;365(20):1876–85.

22. Engeland A, Bjørge T, Tverdal A, Søgaard AJ. Obesity in Adolescence and Adulthood and the Risk of Adult Mortality. Epidemiology. 2004 Jan;15(1):79–85.

23. Pandita A, Sharma D, Pandita D, Pawar S, Tariq M, Kaul A. Childhood obesity: prevention is better than cure. Diabetes Metab Syndr Obes. 2016;9:83–9.

24. Vandenbroeck IP, Goossens J, Clemens M. Foresight Tackling Obesities: Future Choices — Obesity System Atlas. London, UK; 2007.

25. WHO Commission on Ending Childhood Obesity. Ending Childhood Obesity. Geneva; 2016.

26. Spruijt□Metz D. Etiology, treatment, and prevention of obesity in childhood and adolescence: A decade in review. J Res Adolesc. 2011;21(1):129–52.

27. Biro FM, Wien M. Childhood obesity and adult morbidities. Am J Clin Nutr. 2010 May;91(5):1499S–1505S.

28. Hill JO, Wyatt HR, Peters JC. Energy balance and obesity. Circulation. 2012 Jul 3;126(1):126–32.

29. National Institute for Health and Care Excellence (NICE). Lifestyle weight management services for overweight or obese children and young people overview - NICE Pathways [Internet]. Available from: https://pathways.nice.org.uk/pathways/lifestyle-weight-management-services-for-



For peer review only - http://bmjopen.bmj.com/site/about/guidelines.xhtml

overweight-or-obese-children-and-young-people#content=view-node%3Anodes-developing-a-tailored-plan

30. HM Government. Childhood Obesity A Plan for Action. London, UK; 2016.

31. Centers for Disease Control and Prevention (CDC). Nutrition, Physical Activity, and Obesity Prevention Strategies [Internet]. Prevention Strategies & Guidelines. Available from: https://www.cdc.gov/obesity/resources/strategies-guidelines.html

32. Government of Canada. Curbing Childhood Obesity A Federal, Provincial and Territorial Framework for Action to Promote Healthy Weights. Government of Canada; 2010.

33. European Commission - High Level Group on Nutrition and Physical activity. EU Action Plan on Childhood Obesity 2014-2020. Brussels, Belgium: European Commission; 2014.

34. Connelly JB, Duaso MJ, Butler G. A systematic review of controlled trials of interventions to prevent childhood obesity and overweight: a realistic synthesis of the evidence (Structured abstract). Public Health. 2007 Jul;121(7):510–7.

35. Wickremasinghe D, Kuruvilla S, Mays N, Avan BI. Taking knowledge users' knowledge needs into account in health: an evidence synthesis framework. Health Policy Plan. 2016 May;31(4):527–37.

36. Arksey H, O'Malley L. Scoping studies: towards a methodological framework. Int J Soc Res Methodol. 2005 Feb;8(1):19–32.

37. Burrows T, Golley RK, Khambalia A, McNaughton SA, Magarey A, Rosenkranz RR, et al. The quality of dietary intake methodology and reporting in child and adolescent obesity intervention trials: a systematic review. Obes Rev. 2012 Dec;13(12):1125–38.



For peer review only - http://bmjopen.bmj.com/site/about/guidelines.xhtml

38. Khambalia AZ, Dickinson S, Hardy LL, Gill T, Baur LA. A synthesis of existing systematic reviews and meta-analyses of school-based behavioural interventions for controlling and preventing obesity. Obes Rev. 2012 Mar;13(3):214–33.

39. Mallonee LF, Boyd LD, Stegeman C. A scoping review of skills and tools oral health professionals need to engage children and parents in dietary changes to prevent childhood obesity and consumption of sugar-sweetened beverages. J Public Health Dent. 2017 Jun;77 Suppl 1:S128–35.

40. Rivera J, McPherson A, Hamilton J, Birken C, Coons M, Iyer S, et al. Mobile Apps for Weight Management: A Scoping Review. JMIR mHealth uHealth. 2016 Jul 26;4(3):e87.

41. Waters E, de Silva-Sanigorski A, Burford BJ, Brown T, Campbell KJ, Gao Y, et al. Interventions for preventing obesity in children. In: Waters E, editor. Cochrane Database of Systematic Reviews. Chichester, UK: John Wiley & Sons, Ltd; 2011.

42. de Melo Boff R, Arauje Liboni RP, de Azvedo Batista IP, Heineck de Souza L, da Silva Oliveira M. Weight loss interventions for overweight and obese adolescents: a systematic review. Eat Weight Disord. 2017;22:211–29.

43. Al-Khudairy L, Loveman E, Colquitt JL, Mead E, Johnson RE, Fraser H, et al. Diet, physical activity and behavioural interventions for the treatment of overweight or obese adolescents aged 12 to 17 years. In: Rees K, editor. Cochrane Database of Systematic Reviews. Chichester, UK: John Wiley & Sons, Ltd; 2017.

44. Mead E, Brown T, Rees K, Azevedo LB, Whittaker V, Jones D, et al. Diet, physical activity and behavioural interventions for the treatment of overweight or obese children from the age of 6 to 11 years. Ells LJ, editor. Vol. 2017, Cochrane Database of Systematic Reviews. Chichester, UK: John Wiley & Sons, Ltd; 2017.



For peer review only - http://bmjopen.bmj.com/site/about/guidelines.xhtml

45. Levac D, Colquhoun H, O'Brien KK. Scoping studies: advancing the methodology. Implement Sci. 2010;5:69.

46. The Joanna Briggs Institute. The Joanna Briggs Institute Reviewers' Manual 2015 - Methodology for JBI Scoping Reviews. Adelaide, South Australia; 2015.

**FUNDING STATEMENT**

PBVB and GB were funded by the Department of Management at the London School of Economics and Political Science. CDP was funded by the National Institute for Health Research Collaboration for Leadership in Applied Health Research and Care (NIHR CLAHRC) North Thames at Barts Health NHS Trust. The views expressed are those of the authors and not necessarily those of the NHS, the NIHR or the Department of Health.

**COMPETING INTERESTS STATEMENT**

None declared

**AUTHORS' CONTRIBUTIONS**

PBVB contributed to develop the research questions and the methods and contributed substantially to the drafting and editing. CDP conceived of the idea the scoping review, developed the research questions and contributed to the development of the methods. She contributed extensively to the drafting and editing of the manuscript. GB supervised the preparation of the protocol and critically reviewed the manuscript. All Authors have approved the final manuscript.



For peer review only - http://bmjopen.bmj.com/site/about/guidelines.xhtml

**DATA SHARING STATEMENT**

No additional data are available.

For peer review only



For peer review only - http://bmjopen.bmj.com/site/about/guidelines.xhtml

# Supplementary material 1

## Search strategy

### CINAHL Plus via EBSCOhost

| | |
|---|---|
| **Date of search** | 18/08/2017 |
| **Search string** | (TI ( obesity OR overweight OR weight ) OR MH (obesity OR pediatric obesity)) AND (TI ( scoping OR systematic OR meta OR review ) OR MH (review OR meta-analysis)) AND (AB ( child* OR adolescen* OR you* ) OR MH (child OR adolescent)) AND AB ( prevention OR intervention ) |
| **Number of results** | 178 for AB; 185 for SU |
| **Number of results** | 363 |

### Cochrane Library of Systematic Reviews

| | |
|---|---|
| **Date of search** | 18/08/2017 |
| **Search string** | MeSH descriptor: [Pediatric Obesity] explode all trees |
| **Number of results** | 12 |

### ERIC

| | |
|---|---|
| **Date of search** | 26/07/2017 |
| **Search string** | (title:(obesity OR overweight OR weight) AND (title:(scoping OR systematic OR meta OR review)) AND (abstract:(child* OR adolescen* OR you*)) |
| **Number of results** | 454 |

### ERIC via EBSCOhost

| | |
|---|---|
| **Date of search** | 26/07/2017 |
| **Search string** | TI ( obesity OR overweight OR weight ) AND TI ( scoping OR systematic OR meta OR review ) AND AB ( child* OR adolescen* OR you* ) AND AB ( prevention OR intervention ) |
| **Number of results** | 35 |

### Google Scholar

| | |
|---|---|
| **Date of search** | 26/07/2017 |
| **Search string** | (obesity OR overweight OR weight) (scoping OR systematic OR meta OR review) ( child OR adolescen OR you ) (prevention OR intervention)<br>allintitle: (obesity OR overweight OR weight) (scoping OR systematic OR meta OR review) (child OR adolescen OR you) |
| **Number of results** | 104 |

For peer review only



For peer review only - http://bmjopen.bmj.com/site/about/guidelines.xhtml

**Joanna Briggs Institute (JBI) Database of Systematic Reviews and Implementation Reports via OVID**

| | |
|---|---|
| **Date of search** | 28/07/2017 |
| **Search string** | (Ti: ((obesity OR overweight OR weight) AND (scoping OR systematic OR meta OR review))) AND (ab:((child* OR adolescen* OR you*) AND (prevention OR intervention)) |
| **Number of results** | 31 |

**MEDLine/PubdMed MeSH**

| | |
|---|---|
| **Date of search** | 18/08/2017 |
| **Search string** | ((((obesity[Title] OR overweight[Title] OR weight[Title] OR obesity[MeSH Terms] OR pediatric obesity[MeSH Terms])) AND (scoping[Title] OR systematic[Title] OR meta[Title] OR review[Title] OR Review[MeSH Terms] OR Meta-analysis[MeSH-Terms])) AND (child*[Title/Abstract] OR adolescen*[Title/Abstract] OR you*[Title/Abstract] OR Child[MeSH Terms] OR Adolescent[MeSH Terms])) AND (prevention[Title/Abstract] OR intervention[Title/Abstract]) |
| **Number of results** | 792 |

**NHS EED**

| | |
|---|---|
| **Date of search** | 28/07/2017 |
| **Search string** | ((obesity OR overweight OR weight)):TI OR ((scoping OR systematic OR meta OR review)):TI OR ((child* OR adolescen* OR you*)):TI AND ((prevention OR intervention)):TI |
| **Number of results** | 99 |

**PsycINFO via EBSCOhost**

| | |
|---|---|
| **Date of search** | 28/07/2017 |
| **Search string** | TI ( obesity OR overweight OR weight ) AND TI ( scoping OR systematic OR meta OR review ) AND AB ( child* OR adolescen* OR you* ) AND AB ( prevention OR intervention ) |
| **Number of results** | 275 |

**Scopus**

| | |
|---|---|
| **Date of search** | 28/07/2017 |
| **Search string** | TITLE( obesity OR overweight OR weight ) AND TITLE( scoping OR systematic OR meta OR review ) AND ABS( child* OR adolescen* OR you* ) AND ABS( prevention OR intervention ) |



For peer review only - http://bmjopen.bmj.com/site/about/guidelines.xhtml

| | |
|---|---|
| **Number of results** | 501 |

**SocINDEX via EBSCOhost**

| | |
|---|---|
| **Date of search** | 28/07/2017 |
| **Search string** | TI ( obesity OR overweight OR weight ) AND TI ( scoping OR systematic OR meta OR review ) AND AB ( child* OR adolescen* OR you* ) AND AB ( prevention OR intervention ) |
| **Number of results** | 20 |

**Web of Science / Web of Knowledge**

| | |
|---|---|
| **Date of search** | 18/07/2017 |
| **Search string** | TI=((obesity OR overweight OR weight) AND (scoping OR systematic OR meta OR review)) AND TS=((child* OR adolescen* OR you*) AND (prevention OR intervention)) |
| **Number of results** | 567 |



For peer review only - http://bmjopen.bmj.com/site/about/guidelines.xhtml

For peer review only

# Supplementary material 2

## PRIMA flowchart

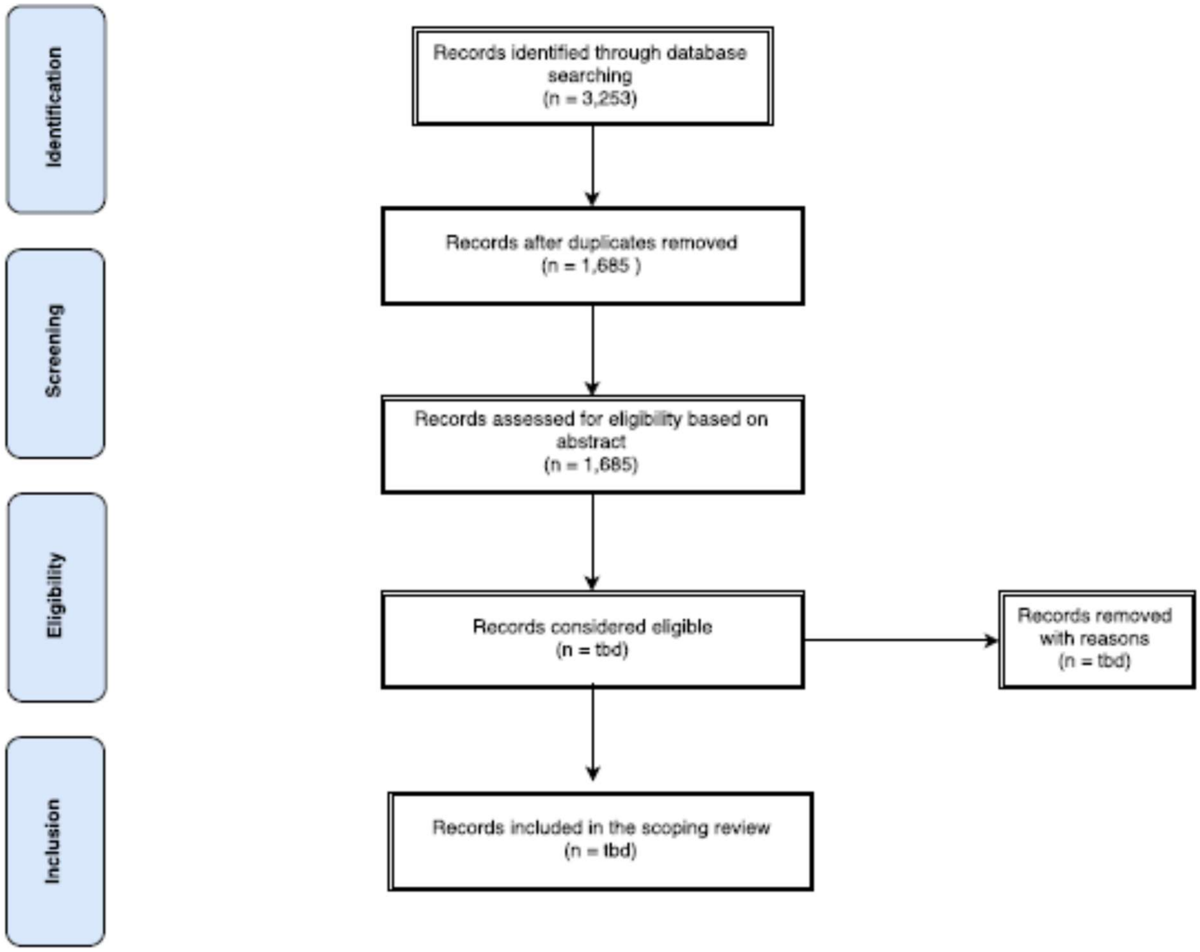

Supplement: Author's manuscript [file bmjopen-2017-019311.draft_revisions.pdf]
